# Duodenal obstruction due to accidental swallowing of a dental prosthesis: a case report and review of the literature

**DOI:** 10.1186/s13256-020-02456-z

**Published:** 2020-08-15

**Authors:** Saadat Mehrabi, Mohammad Javad Yavari Barhaghtalab, Reza Hosseinpour

**Affiliations:** Department of General Surgery, Shahid Beheshti Hospital, Yasuj University of Medical Sciences, Yasuj, Iran

**Keywords:** Artificial denture, Duodenal obstruction, Perforation, Endoscopy, Gastrostomy

## Abstract

**Background:**

Artificial dentures are the most common object ingested by elderly patients and account for 4–18% of all foreign body ingestions. Denture impaction in the small bowel is a rare phenomenon. Surgery of the duodenum is difficult, so endoscopy should be the first choice in these patients. There are very rare case reports on denture ingestion-induced duodenal obstruction or perforation, so the aim of this publication was to show a rare case of accidental ingestion of a dental prosthesis with duodenal obstruction and also perforation that could not be treated with endoscopic management and was managed with duodenal kocherization and gastrostomy.

**Case presentation:**

A 47-year-old Iranian woman presented to our hospital with epigastric abdominal pain of 2 hours’ duration after the accidental ingestion of a dental prosthesis 2 days before admission. The patient had severe epigastric tenderness. Radiographic examination revealed nothing. Upper gastrointestinal endoscopy showed a swallowed lodged denture in the second to third parts of the duodenum, and the attempt to extract the denture failed. The patient underwent laparotomy and duodenal kocherization, pushing the denture to the stomach, and gastrostomy, and the denture was brought out without any complications.

**Conclusions:**

Patients with old and worn dentures should have their prosthesis reconstructed and redesigned periodically in order to prevent denture ingestion and its complications. Early surgical intervention is recommended in patients with failed endoscopic extraction of foreign bodies and in those with duodenal perforation.

## Background

Ingested foreign bodies progress through the digestive tract spontaneously in 80–90% of cases; however, 10–20% of patients require endoscopy for removal, and less than 1% undergo surgery [1]. Ingested foreign bodies are commonly seen in patients with alcohol overuse and drug misuse due to impaired judgment; emotional disturbance; psychiatric disorders such as schizophrenia, mental retardation, and Alzheimer disease; and in persons who wear artificial dentures [2].

According to the current literature, the frequency of swallowed foreign bodies in adults varies widely. The more commonly swallowed foreign bodies among adults are fish bones (9–45%), bones other than fish bones (8–40%), and dentures (4–18%) [3]. Dentures impacted in different parts of the gastrointestinal (GI) tract lead to various surgical complications, including perforation, bleeding, and obstruction [4, 5]. Patients with prior abdominal surgery, acute angulation, physiological narrowing in the GI tract, or congenital gut malformations are at an increased risk for such complications. Risk factors that increase the probability of perforation include the presence of intrinsic bowel diseases, such as adhesions, inflammatory bowel disease, tumors, diverticula, hernia, or blind segments [6]. Endoscopy can be used for the extraction of swallowed artificial dentures, but in a number of cases, endoscopy fails, which leads the physician to plan surgical exploration and removal [7, 8].

There are very rare case reports on denture ingestion-induced duodenal obstruction and perforation, and we found six previous reports to use in our review of the literature, as shown in Table 1 [9–14]. The aim of this study was to report a rare case of accidental ingestion of a dental prosthesis with duodenal obstruction and perforation that could not be treated with endoscopic management and was managed with duodenal kocherization and gastrostomy.

**Table 1:** Review of literature on duodenal obstruction or perforation after ingestion of denture

Case	Country, year of study [reference]	Age (years)	Sex	Risk factors	Comorbid diseases	Chief complaints	Physical examination	Abdominal radiology	Endoscopy for extraction	Location	Complications	Surgery
1	Japan, 2003 [9]	82	Male	Not mentioned	BPH	No discharged ingested denture for 3 days	Mild tenderness in epigastric area	Serial x-rays showed that the denture didn't move forwards.	Performed without success	Horizontal part of duodenum	Perforation	Duodenotomy
2	India, 2006 [10]	59	Male	Not mentioned	Not mentioned	Pain in the right upper abdomen after accidental swallowing of a denture 2 weeks earlier	Tender, firm, and fixed lump measuring 6 × 8 cm in right hypochondrium with smooth surface	X-ray was not mentioned; CT scan revealed pathology in duodenum	Ulceration in first part of duodenum	Third part of duodenum	Necrotic mass in mesentery of thickened third part of duodenum (penetration)	Not done (surgical exploration was advised, but patient refused)
3	Japan, 2010 [11]	49	Male	Mental retardation	Not mentioned	Abdominal pain	Supraumbilical abdominal tenderness + high-grade fever	X-ray showed radiopaque object; CT scan showed foreign body in duodenum + free air and fluid collection in retroperitoneal space around duodenum	Not performed	Posterior wall of duodenum	Perforation	Laparotomy, closure of perforation, cholecystectomy, T-tube drainage, and gastrostomy, followed by intraperitoneal irrigation and drain placement near perforation
4	Turkey, 2011 [12]	33	Male	Schizophrenia, poor oral and dental hygiene	Negative	Acute abdominal pain, bilious vomit, and nausea	Mild abdominal tenderness	X-ray showed radiopaque object	Performed without success	Third part of duodenum	Obstruction	Gastrostomy
5	Pakistan, 2017 [13]	63	Male	Senile dementia, poorly fitting dentures, and poor oral and dental hygiene	COPD, musculoskeletal	Acute abdominal pain	Abdominal distension + generalized guarding	X-ray showed air under the diaphragm + radiopaque object in upper right quadrant of abdomen	Not performed	Second part of duodenum	Obstruction, perforation, and frank peritonitis	Duodenotomy + feeding jejunostomy
6	China, 2019 [14]	69	Male	Alzheimer disease	Not mentioned	Dysphagia, epigastric pain, bilious vomiting, and severe nausea	No pathological findings	Irregular densification in right middle abdomen; CT scan showed prosthesis	Performed with success	Descending part of duodenum	Impaction of denture in duodenum	Not done (successfully brought out with endoscopy)
7	Iran, 2019 (our patient)	47	Female	Old dental prosthesis and poor oral and dental hygiene	Asthma, migraine headache	Epigastric abdominal pain, nausea, vomiting, and anorexia	Severe epigastric and mild right upper quadrant abdominal tenderness	X-ray showed nothing; CT scan revealed pathology in duodenum	Performed without success	Second and third parts of duodenum	Both obstruction and perforation	Gastrostomy and duodenal kocherization

*Abbreviations: COPD* Chronic obstructive pulmonary disease, *CT* Computed tomography, *BPH *Benign prostatic hyperplasia

## Case presentation

**Fig. 1:**
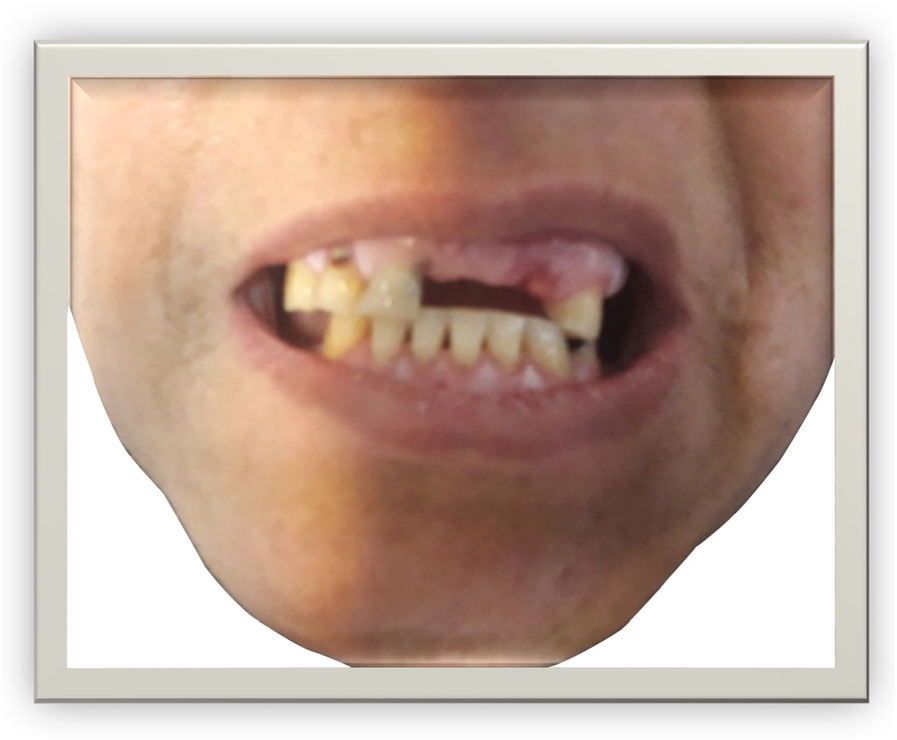
Picture of our patient with loss of upper anterior dentures

A 47-year-old Iranian woman presented to our hospital with the chief complaint of pain in the abdomen (mostly in the epigastric area) for the previous 2 days, associated with recurrent vomiting, nausea, and anorexia. Her complaints had started 2 hours after the accidental ingestion of a dental prosthesis about 2 days before admission (Fig. 1). The patient has a known history of asthma and migraine headache and a history of wearing artificial dentures. At the time of admission, she was conscious and oriented to time, place, and person. Her pulse rate was 90 beats per minute, and her blood pressure was 100/60 mmHg. Her physical examination revealed severe epigastric and mild right upper quadrant abdominal tenderness.

**Fig. 2:**
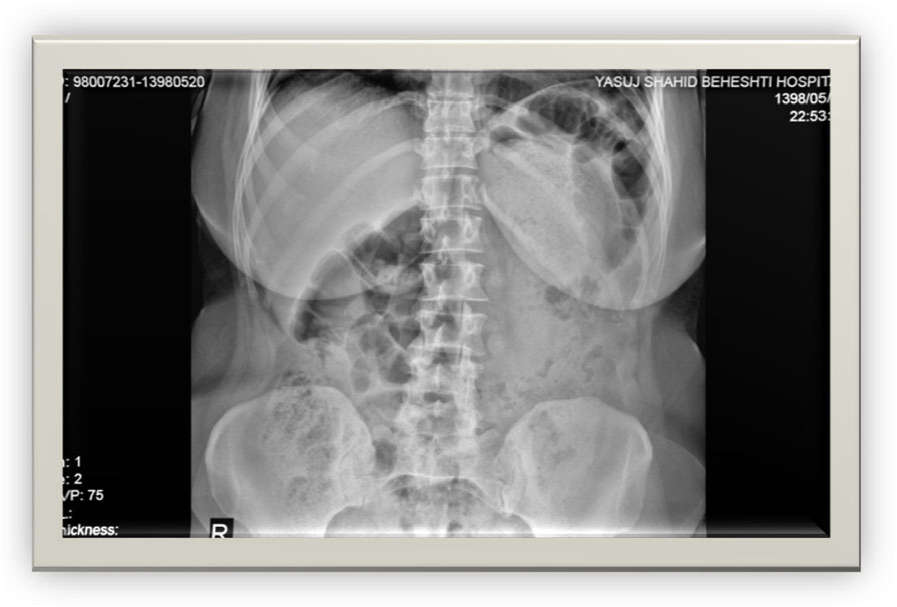
Normal upright abdominal x-ray

**Fig. 3:**
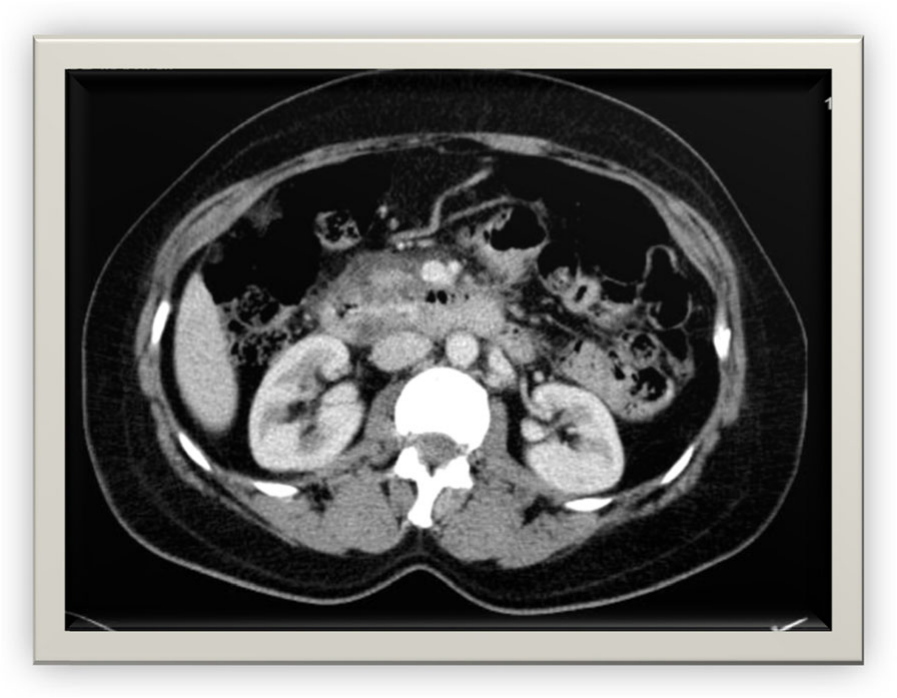
Wall thickness, peripheral mesenteric fat edema, hematoma, and narrowing of the duodenum

**Fig. 4:**
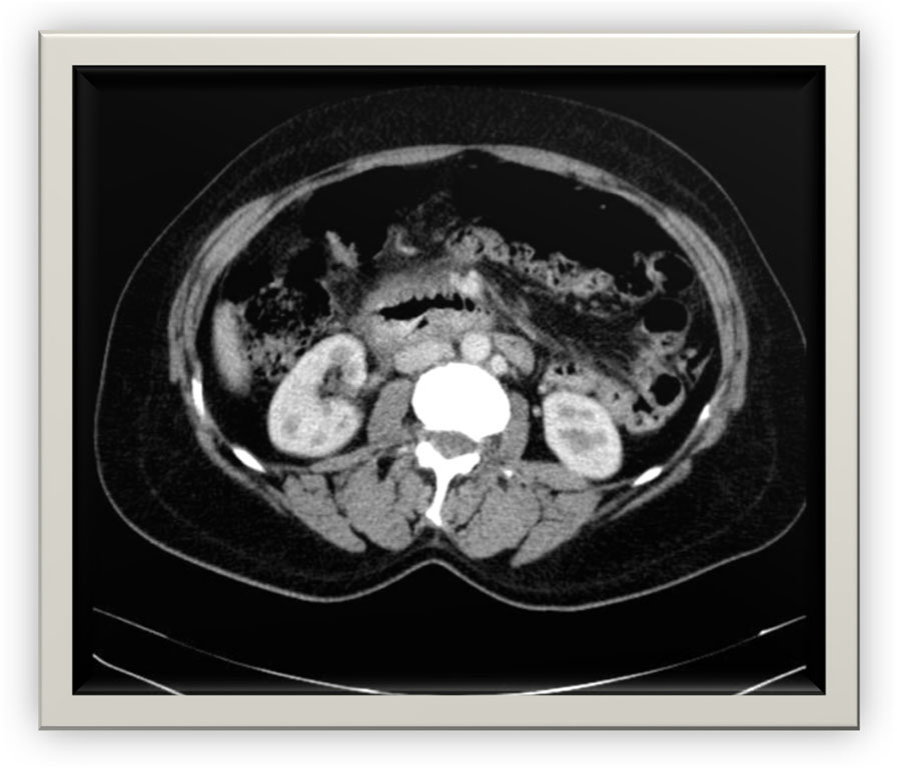
Air in intestinal wall, pneumoretroperitoneum, laceration, and microperforations in the duodenum

A radiograph showed no free gas under the right dome of the diaphragm and no air-fluid level (Fig. 2). Ultrasonography revealed that there was no free fluid in the peritoneal cavity. Spiral abdominal computed tomography (CT) with oral and intravenous contrast revealed duodenal wall thickness in D2–4, peripheral mesenteric fat edema, hematoma in D1–3, air in the intestinal wall, pneumoretroperitoneum, laceration in D1–2 and a part of D3, and microperforations in D2–3. Because of the hematoma, narrowing in the primary and middle parts of the duodenum was seen (Figs. 3 and 4).

Video endoscopic findings were an old healed linear ulcer scar in the bulb of the duodenum, and also a swallowed lodged denture was seen in D2–3 after a papilla, which left a laceration behind. An attempt to capture the foreign body was not possible because of difficulty in the procedure and the chance of induced laceration, so a gastroenterologist recommended surgical evacuation of the denture. Laboratory investigation results are shown in Table 2.

**Table 2:** Laboratory findings in our patient

Laboratory results	Normal range
WBC 12,500/mm^3^	4300–10,800 cells/mm^3^
Hemoglobin 10.9 g/dl	Female: 12.1–15.1 g/dl
Platelets 254,000/μl	150,000–400,000 platelets/μl
Serum amylase 35 U/L	30–110 U/L
Blood sugar 98 mg/dl	<140 mg/dl
Serum creatinine 0.88 mg/dl	0.7–1.2 mg/dl
Blood urea nitrogen 10 mg/dl	7–20 mg/dl
Urine examination	Normal

*WBC* White blood cells, *cells/mm*^*3*^ Cells per cubic millimeter, *g/dL* Grams per deciliter, *U/L* Units per liter, *mg/dl* Milligrams per deciliter

**Fig. 5:**
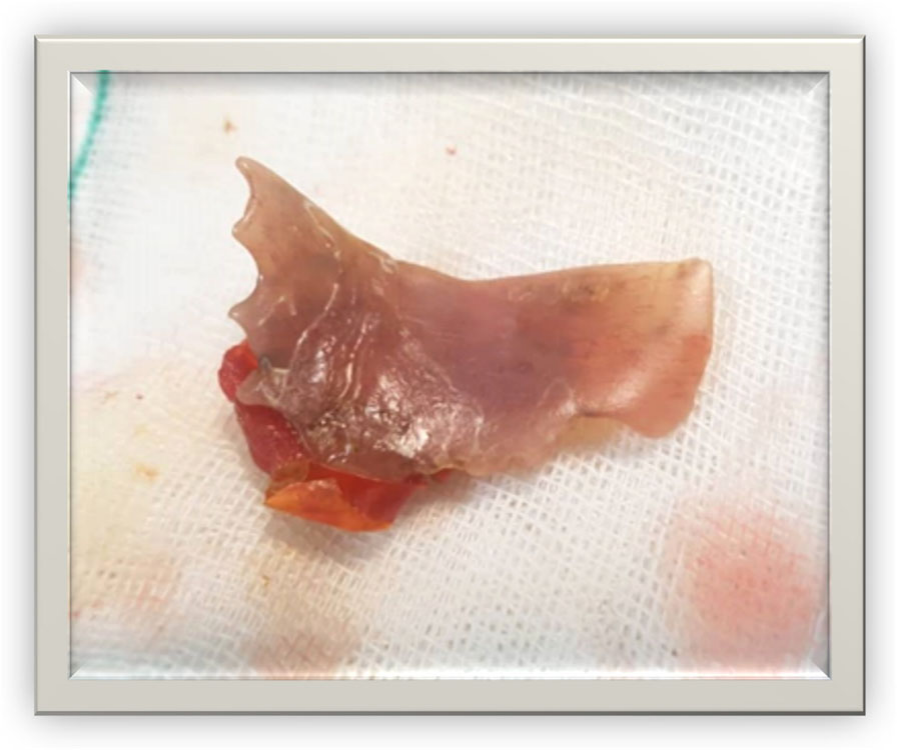
Artificial teeth (radiolucent plastic part known as the polymethylmethacrylate part) with two sharp and two blunt edges and a portion of food material (tomato) attached behind

After preparation of the operation site, a midline laparotomy was performed. The stomach and small bowel were examined carefully for any perforation, but no pathology was revealed; however, a foreign body was palpable between the second and the third portions of the duodenum inside the lumen, so duodenal kocherization was performed to explore the duodenum for any perforations. There was no gross perforation in the duodenal wall, so the foreign body was pushed through the lumen to pass back to the stomach from the pylorus. A gastrostomy was performed, and the foreign body was removed and identified as an artificial denture (Fig. 5). Then the stomach was repaired in two layers. Washing with normal saline was done, and an abdominal drain was placed at the duodenal site of kocherization. The patient recovered well postoperatively and was discharged on the ninth day of her total hospitalization in good condition.

## Discussion and conclusions

Most reported cases of accidental swallowing of dentures are related to food ingestion [13]. The anatomic sites of obstruction in descending order are the upper esophageal sphincter (cricopharyngeal area), the aortic arch, the lower esophageal sphincter (diaphragmatic hiatus), the pylorus, and the ileocecal valve [15, 16]. Once the object reaches the stomach, it can continue to pass through to the ileocecal region almost without difficulty [4]. Denture impaction in the small bowel is a rare phenomenon [4]. The passage through the duodenum depends on the diameter as well as the length of the ingested foreign body. Foreign bodies with lengths more than 6 cm and diameters more than 2.5 cm pass the duodenum with difficulty [3]. The base of an artificial denture is made of a radiolucent plastic material known as polymethylmethacrylate (Fig. 5), whereas the tooth is made of porcelain. The radiopaque area of a denture is the metal pin that holds it together. A multiplanar CT scan is the preferred choice to identify the exact location of radiolucent dentures [13]. A dislodged swallowed denture usually presents within a few days and can be followed successfully passing through the GI tract to the rectal canal by serial radiological investigations [13]. If an object remains motionless for 3 days in the intestine or for 1 week in the stomach, surgery is required [17]. Surgery of the duodenum is difficult, so endoscopy should be the first choice for patients in whom a foreign object is demonstrated to be fixed in the upper GI system. In cases in which endoscopic extraction fails, surgery should be considered [12].

In our patient’s case, endoscopy failed to bring out the swallowed denture, which led us to perform surgical exploration and removal. Our review of the literature on duodenal obstruction or perforation after ingestion of dentures found six previous studies. Table 1 provides a summary of specific characteristics of these studies and also our present case report, including demographic and risk factors, chief complaints, physical examinations, abdominal radiological findings, endoscopy for extraction of the denture if done, location of obstruction or perforation, and kind of surgery. According to our report and the six previous reports mentioned [9–14], if there is evidence of duodenal perforation or if the endoscope is unable to remove the foreign body early, surgical intervention is recommended.

Our present case report is different from other case reports in the literature according to the patient’s sex; our patient was female, but none of the earlier cases reported were female. Our patient had both obstruction and perforation, similar to the patient in the Siddiq *et al.* [13] study, but our patient’s case was comparable to other case reports in which only one of obstruction, perforation, impaction, and penetration was the dominant pathology [9–14]. This process might be due to excessive dilation of the duodenum due to the progression of bowel obstruction, which results in fragility of the intestinal wall and leads to perforation and even the generation of peritonitis [18].

Artificial dentures are the most common object ingested by elderly patients. However, there have been reports that accidental ingestion of foreign bodies is increasing because of an aging society in recent years, and accidental ingestion of dentures has increased by about twofold. The larger and sharper the denture, the more complications occur. In cases in which endoscopic extraction fails, surgery should be considered. During surgery, attention must be paid not to harm the duodenum. Patients with old and worn dentures should have their prostheses reconstructed and redesigned periodically in order to prevent denture ingestion and its complications. Early surgical intervention is recommended in patients with failed endoscopic extraction of foreign bodies and in those with duodenal perforation.

## Data Availability

All data generated or analyzed during this study are included in this article.

## Abbreviations

CT: Computed tomography
GI: Gastrointestinal
WBC: White blood cells
